# *N*-Acetyl-l-leucine improves functional recovery and attenuates cortical cell death and neuroinflammation after traumatic brain injury in mice

**DOI:** 10.1038/s41598-021-88693-8

**Published:** 2021-04-29

**Authors:** Nivedita Hegdekar, Marta M. Lipinski, Chinmoy Sarkar

**Affiliations:** 1 Department of Anesthesiology, Shock, Trauma and Anesthesiology Research (STAR) Center, University of Maryland School of Medicine, Baltimore, MD 21201 USA; 2 Department of Anatomy and Neurobiology, University of Maryland School of Medicine, Baltimore, MD 21201 USA

**Keywords:** Drug discovery, Neuroscience, Diseases, Neurology

## Abstract

Traumatic brain injury (TBI) is a major cause of mortality and long-term disability around the world. Even mild to moderate TBI can lead to lifelong neurological impairment due to acute and progressive neurodegeneration and neuroinflammation induced by the injury. Thus, the discovery of novel treatments which can be used as early therapeutic interventions following TBI is essential to restrict neuronal cell death and neuroinflammation. We demonstrate that orally administered *N*-acetyl-l-leucine (NALL) significantly improved motor and cognitive outcomes in the injured mice, led to the attenuation of cell death, and reduced the expression of neuroinflammatory markers after controlled cortical impact (CCI) induced experimental TBI in mice. Our data indicate that partial restoration of autophagy flux mediated by NALL may account for the positive effect of treatment in the injured mouse brain. Taken together, our study indicates that treatment with NALL would be expected to improve neurological function after injury by restricting cortical cell death and neuroinflammation. Therefore, NALL is a promising novel, neuroprotective drug candidate for the treatment of TBI.

## Introduction

Traumatic brain injury (TBI) is a major health concern all over the world. Between 1990 and 2016, the prevalence of TBI increased by around 8.4%^1^. Almost 27 million new cases of TBI were reported worldwide in 2016, with an incidence rate of 369 per 100,000 people^1^. In the US around 1.7 million cases of TBI are reported each year^2,3^. People affected by brain injuries can suffer from lifelong physical disabilities, cognitive decline and premature death due to severe and progressive neurodegeneration and neuroinflammation^2,4,5^. TBI patients are also at high risk of developing neurodegenerative diseases such as Alzheimer’s disease and Parkinson’s disease later in life^6,7^. TBI often results in severe social, emotional and financial distress to the patients, their families and caregivers as well as puts a significant financial burden on society^3^, indicating the need for development of more effective therapeutic strategies to restrict brain damage after TBI.

In TBI, primary mechanical injury to the brain, for example due to a fall or a vehicle accident, triggers a cascade of secondary deleterious events that can propagate the injury and last anywhere from days to years following the original traumatic impact. These include excitotoxicity, perturbation in calcium homeostasis, oxidative stress and activation of caspases and calpain, leading to acute and progressive neurodegeneration^5^. TBI also induces highly complex inflammatory responses that include rapid proliferation of resident microglial cells and infiltration of peripheral macrophages and other monocytes into the injured brain^8–10^. These activated immune cells release a variety of proinflammatory cytokines that can become neurotoxic and further contribute to both acute and chronic neurodegeneration^5,8,9^. While the primary mechanical injury in TBI is instantaneous and cannot be altered, the secondary injury’s extended timeframe represents a therapeutic window for a treatment aimed at restricting neuronal cell death and suppressing neuroinflammation after TBI.

In clinical and pre-clinical studies it has been shown that *N*-acetyl-leucine (NAL), an acetylated derivative of leucine, improves neurological function in cerebellar ataxias^11,12^. The racemic mixture, *N*-acetyl-dl-leucine (dl-NAL) has been used as a medication for the treatment of acute vertigo and vertiginous symptoms in France since 1957; it is orally available and has a well-established safety profile^13^. Electrophysiological studies in a guinea pig model of acute unilateral vestibulopathy demonstrated that DL-NAL can restore the membrane potential of abnormally polarized neurons of the medial vestibular nucleus^14^. In a rat model of an acute unilateral vestibular lesion *N*-acetyl-l-leucine stereospecifically (i.e. not the d-enantiomer, see below) improved central compensation of postural symptoms in a dose-dependent manner^15^. More recent studies in the mouse model of Niemann-Pick disease type C (NPC)—a neurodegenerative lysosomal storage disorder caused by mutation in cholesterol transporting NPC 1 and 2 genes—identified that in addition to symptomatic effects, treatment with the l-enantiomer has a neuroprotective, disease-modifying effect^16,17^. Promising clinical outcomes following racemic NAL treatment, including improved ataxic symptoms and stabilized disease progression, were observed among small cohort of NPC patients, correlating to the pharmacological action of the drug observed in animal studies^16,18,19^. These reports led us to hypothesize that NAL may be useful in improving outcomes after TBI.

The enantiomers of NAL are pharmacologically different and exert distinct toxicity due to their unique pharmacokinetic properties. It has been previously observed that the l-enantiomer (NALL) is the pharmacologically active enantiomer of the racemate responsible for the long-term, neuroprotective, disease modifying effects^16^. Administration of d-isomer of NAL was found to be ineffective compared to its l-form in reducing neuroinflammation or correcting relative lysosomal volume in Niemann-Pick disease type C, consistent with the d-isomer having no neuroprotective effect^16^. As mentioned above, the l-isomer was also reported to be the only active form of NAL that improves functional recovery after vestibular neurectomy in the rat and cat^15,20^. In addition, differences in the pharmacokinetics of NAL enantiomers indicate chronic administration of the racemate could have negative effects and support the isolated use of NALL^21^. Based on the superiority of the l-enantiomer, three clinical trials are ongoing with NALL for the treatment of NPC, GM2 gangliosidosis, and ataxia telangiectasia^22^ (clinicaltrials.gov NCT03759639, NCT03759665, NCT03759678, and EudraCT 2018-004331-71; 018-004406-25; 2018-004407-39).

Accordingly, in this study, we assessed whether NALL is effective in preventing neurodegeneration and neuroinflammation after controlled cortical impact (CCI) induced experimental TBI in mice. Our data demonstrate that treatment with oral NALL can attenuate cell death after TBI in a controlled cortical impact (CCI) mouse model. This is associated with a decrease in several neuroinflammatory markers as well as improvement in autophagy flux. We also observed improved recovery of motor and cognitive function and reduction in lesion volume in injured mice treated with NALL. Together, these data provide further evidence of neuroprotective effect of NALL and indicate NALL as a promising novel drug candidate for the treatment of TBI.

## Results

### NALL treatment does not negatively affect food intake and body weight of mice after TBI

**Figure 1:**
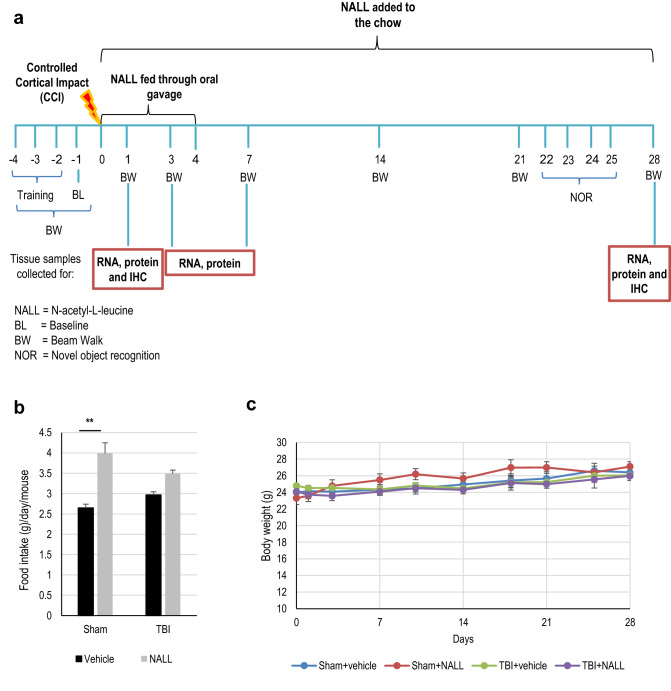
NALL does not affect food intake and body weight in mice. (**a**) NALL treatment strategy. Mice were orally fed with NALL and cortical tissues were collected at days 1, 3 and 7 after TBI for biochemical analyses. *BW* beam walk, *NOR* novel object recognition, *BL* Baseline (**b**) Amount of NALL containing chow eaten by the mice. (**c**) Body weight of sham and injured mice fed with vehicle or NALL. No significant differences were observed between corresponding vehicle and NALL groups. Data are presented as mean ± SEM. n = 10 sham + vehicle, 9 sham + NALL, 20–25 TBI + vehicle and 18 TBI + NALL. **p < 0.001 (Two-way ANOVA with Sidak posttests).

To investigate if NALL treatment can attenuate secondary injury and improve recovery after TBI, we orally administered NALL to C57/BL6 mice for 1–28 days following controlled cortical impact (CCI) induced experimental TBI or sham surgery as depicted in Fig. 1a. Food intake was slightly higher in mice fed with NALL, particularly in the sham group (p < 0.001) (Fig. 1b). While a slight decrease in body weight was detected among all TBI mice (both vehicle and NALL-treated) at days 1 and 3 after injury, they gradually regained the body weight and no appreciable differences in their body weight was observed at day 28 after TBI (mean weight of 26.05 ± 1.99 g in vehicle fed TBI mouse group vs 25.98 ± 2.27 g in NALL-fed TBI mouse group) (Fig. 1c). Body weight increased gradually in both vehicle and NALL fed sham mice over the course of study. These results clearly suggest that inclusion of NALL in chow does not negatively affect food intake or body weight in mice with or without TBI.

### NALL treatment attenuates cortical cell death after TBI

**Figure 2:**
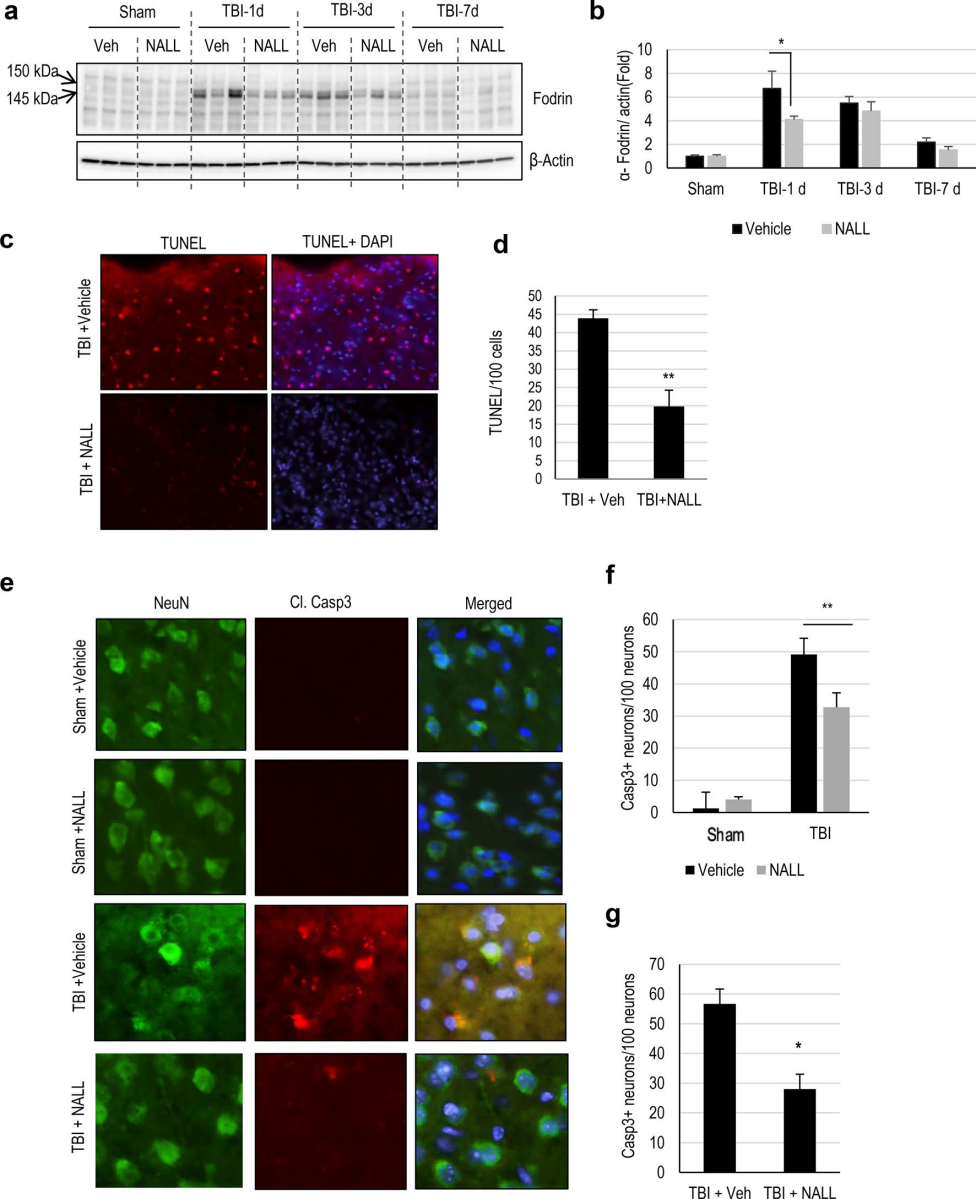
NALL attenuates cortical cell death after TBI. (**a**) Western blot of cortical tissue lysates from sham and TBI mice fed with NALL or vehicle to detect α-fodrin breakdown products. (**b**) Corresponding densitometric analysis of cleaved bands of α-fodrin with respect to β-actin. Data are presented as mean ± SEM. n = 5, *p < 0.05 (Two-way ANOVA with Bonferroni posttests). (**c**) 20 × representative images of vehicle or NALL-fed mouse cortical brain sections at 1 day post- post-TBI stained for TUNEL and (**d**) corresponding quantification. Data are presented as mean ± SEM. n = 5, **p < 0.01 (Two-tailed Student’s t-test). (**e**) 20 × IF representative images of vehicle or NALL-fed mouse cortical brain sections at 1 day post- post-TBI stained for cleaved caspase-3 and NeuN and (**f**) corresponding quantification, represented as number of cleaved caspas-3 + neurons/100 neurons. Data are presented as mean ± SEM. n = 5/group, **p < 0.01 (Two-way ANOVA with Sidak posttests). (**g**) Quantification of cleaved caspase 3 positive neurons in hippocampal brain sections of vehicle or NALL-fed mice at 1 day post-TBI, represented as cleaved caspase-3 + neurons/100 hippocampal neurons. Data are presented as mean ± SEM. n = 5/group *p < 0.05 (Two-tailed Student’s t-test).

CCI is associated with extensive acute cell death peaking at 1 day after injury^23^. We investigated whether NALL treatment can decrease overall cortical cell death after TBI by assessing the level of α-fodrin cleavage products in NALL, vehicle-treated sham, and TBI mouse cortices by western blot. TBI induces both calpain and caspase mediated cleavage of α-fodrin generating 145–150 kDa and 150–120 kDa fragments, respectively^23^. We observed significantly lower level of 145–150 kDa fragments of α-fodrin in the NALL fed mouse cortices as compared to the cortices of mice fed with vehicle at 1 day following TBI, while the levels remained unchanged or slightly lower in the NALL treated groups at the subsequent time points. This indicates the protective benefit of NALL in preventing cortical cell death which peaks at early time points (day 1) after TBI^23^ (Fig. 2a,b and Supplementary Fig. S1). We further investigated the effect of NALL on apoptosis-mediated cortical cell death by TUNEL assay. We detected significantly lower numbers of TUNEL positive cells in the cortical sections of injured mice fed with NALL as compared to vehicle fed TBI mice (Fig. 2c,d). Since, early cortical cell death peaks in neurons after CCI induced TBI in mice^23^; we determined the levels of apoptotic cell death marker—cleaved caspase-3 in the neurons (stained with NeuN) of TBI mice treated with NALL or vehicle. Our data showed significant attenuation of neuronal apoptosis in the cortices of TBI mice treated with NALL as compared to the vehicle treated mice (Fig. 2e,f). Furthermore, we also detected decrease in apoptotic cell death in the hippocampal neurons of TBI mice treated with NALL as compared to the vehicle treated mice (Fig. 2g). These data clearly suggest that NALL treatment is able to attenuate cortical cell death after TBI.

### NALL treatment restores autophagy flux after TBI

**Figure 3:**
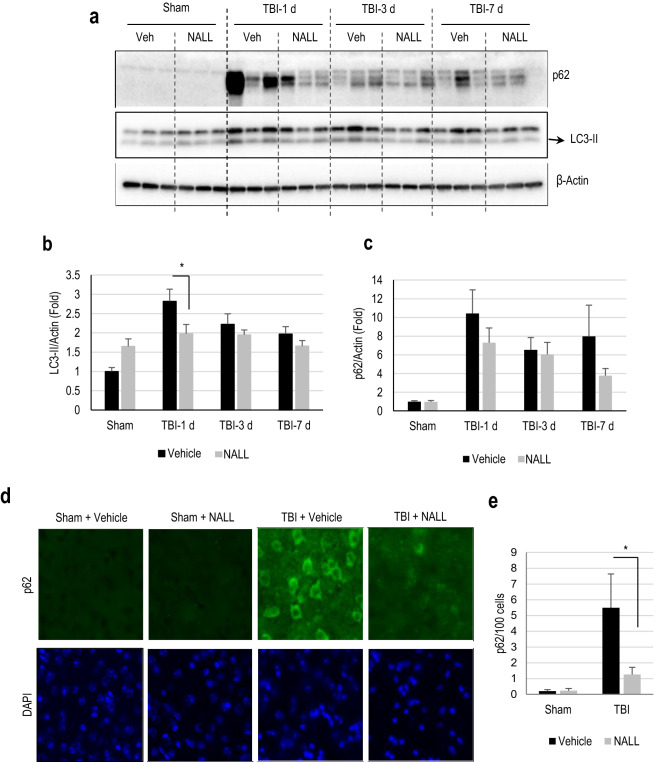
NALL restores autophagy flux after TBI. (**a**) Western blot of cortical tissue lysates from sham and TBI mice fed with NALL or vehicle for autophagosomal marker LC3 and autophagic cargo adaptor proteins p62/SQSTM1 and (**b**,**c**) corresponding densitometric analysis. Data are presented as mean ± SEM. n = 5 (only 3/group included for representative images for western blot analysis), *p < 0.05 (Two-way ANOVA with Bonferroni posttests). (**d**) IF staining for p62/SQSTM1 on frozen sections prepared from mouse cortical brain sections at 1 day after TBI. (**e**) Quantification of image data from d. Data are presented as mean ± SEM. n = 5/group, *p < 0.05 (Two-way ANOVA with Sidak posttests).

Recently, we demonstrated that autophagy, a lysosome dependent cellular degradation process, is disrupted due to lysosomal damage after TBI^23,24^. Autophagy is an important process for neuronal cells as it removes intracellular protein aggregates and damaged organelles^25–27^. We reported that disruption of autophagy flux after TBI causes accumulation of autophagosomal marker LC3-II and autophagic substrate p62/SQSTM1, and is associated with neuronal cell death^23^. Hence, we assessed if the attenuation of cell death following NALL treatment in TBI mice may be associated with the restoration of autophagy flux. The levels of LC3-II and p62/SQSTM1 in the cortical tissue lysates prepared from vehicle and NAL fed sham and TBI mice were determined by western blot. We detected a significant decrease in the levels of LC3-II in the brains of NALL fed mice as compared to the vehicle-treated mice at day 1 after TBI (Fig. 3a,b and Supplementary Fig. S2). We also observed a decreasing trend in p62/SQSTM1 levels in the cortex of mice fed with NALL as compared to the vehicle-treated TBI mice (Fig. 3a,c). We confirmed decrease in p62/SQSTM1 accumulation in NALL-fed TBI mouse cortices as compared to the vehicle-treated TBI controls by immunofluorescence staining (Fig. 3d,e). These data demonstrate that NALL can improve autophagy flux in the mouse cortices after TBI, which may contribute to its neuroprotective function.

### NALL reduces expression of inflammatory cytokines in TBI mouse brain

**Figure 4:**
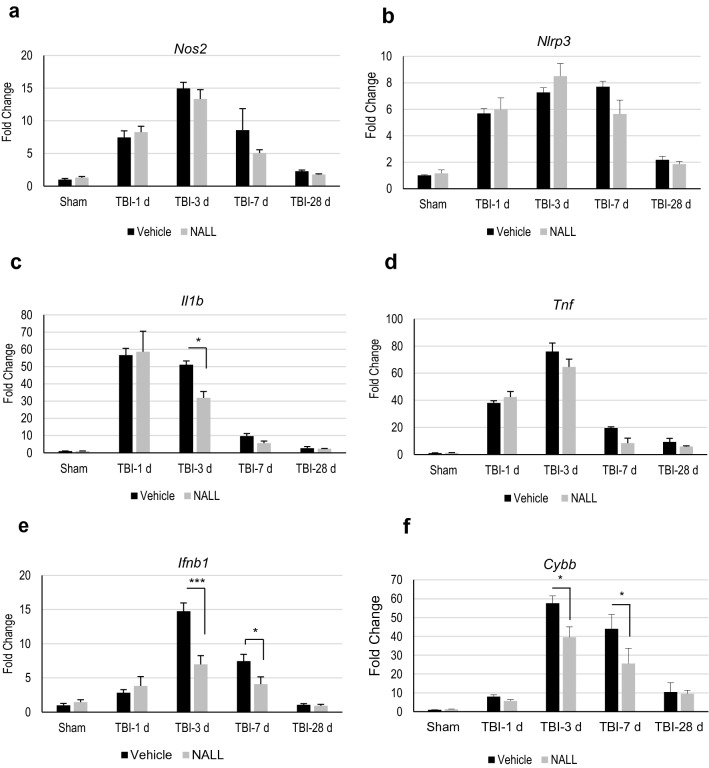
NALL reduces pro-inflammatory markers in the injured mouse cortices. Relative mRNA levels of (**a**) iNOS (*Nos2*), (**b**) NLRP3 (*Nlrp3*), (**c**) IL-1β (*Il1b*), (**d**) TNF (*Tnf*), (**e**) IFN-β (*Ifnb1*) and (**f**) NOX2 (*Cybb*) in the cortices of sham and TBI mice fed with NALL or vehicle. Data are presented as mean ± SEM. n = 5/group, ***p < 0.001, *p < 0.05 (Two-way ANOVA with Bonferroni posttests).

**Figure 5:**
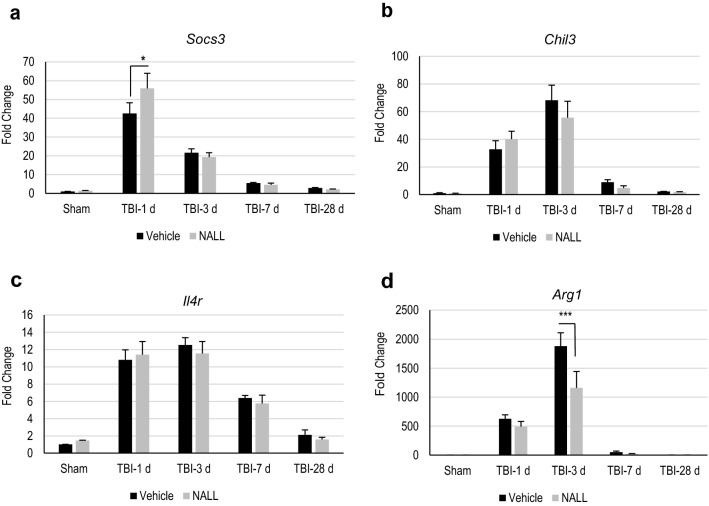
NALL alters the levels of anti-inflammatory markers in the injured mouse cortices. Relative mRNA levels of (**a**) SOCS3 (*Socs3*), (**b**) YM-1 (*Chil3*), (**c**) IL-4R (*Il4r*) and (**d**) Arginase (*Arg1*) in the cortices of sham and TBI mice fed with NALL or vehicle. Data are presented as mean ± SEM. n = 5/group, ***p < 0.001, *p < 0.05 (Two-way ANOVA with Bonferroni posttests).

We examined whether NALL treatment can reduce neuroinflammation in the brain following TBI. We determined mRNA expression level of pro-inflammatory markers iNOS (*Nos2*), Nlrp3 (*Nlrp3*), IL-1β (*Il1b*), TNF (*Tnf*), IFN-B1(*Ifnb1*) and Nox2 (*Cybb*) in the perilesional area in TBI mouse cortices by real time RT-PCR (Fig. 4a–f). We observed elevated levels of all inflammatory markers, starting from day 1 and peaking at day 3 after injury, in all TBI mouse cortices, irrespective of treatment. However, we detected a significant decrease in the mRNA level for *IL1b*, *Ifnb1* and *Cybb* in the cortices of NALL-fed TBI mice as compared to the vehicle fed TBI mouse cortices (Fig. 4c,e,f). A decreasing trend in *Tnf* level in TBI mice fed with NALL as compared to the vehicle fed injured mice was also observed but did not reach significance (Fig. 4d). Similar to pro-inflammatory markers, we observed higher expression of anti-inflammatory markers Socs3 (*Socs3*), YM-1 (*Chil3*), IL4ra (*Il4r*) and Arg-1 (*Arg1*) in the cortical tissue of all TBI mice as compared to sham animals (Fig. 5a–d). Among these markers, NALL treatment significantly increased *Socs3* expression and lowered levels of *Arg1* in the injured mouse cortex as compared to vehicle fed TBI mice (Fig. 5a,d). Taken together, these results demonstrate that NALL reduces expression of several inflammatory markers, thus indicating overall decrease in neuroinflammation following TBI in mice.

### NALL treatment improves motor and cognitive function in mice after TBI

**Figure 6:**
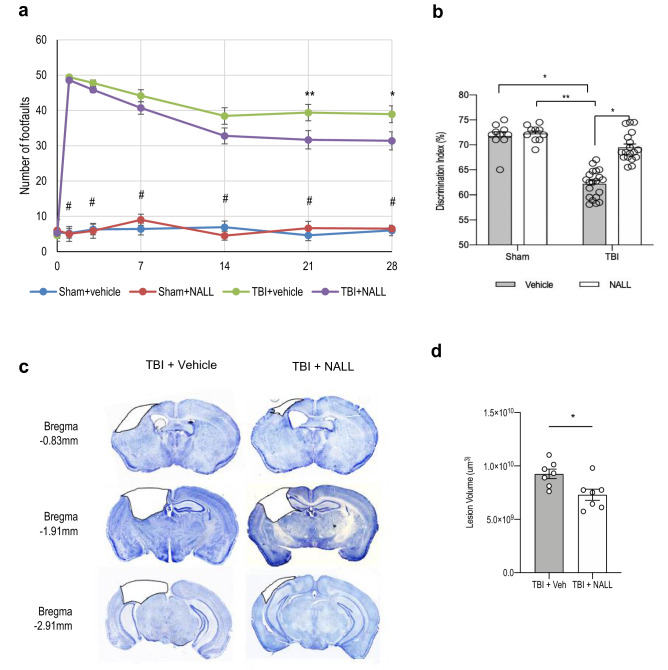
NALL attenuates motor and cognitive deficits and cortical tissue loss in mice after injury. (**a**) Sensory motor function was assessed in sham and TBI mice treated with vehicle or NALL using beam walk test. Number of hindlimb foot faults per 50 steps was counted on days 0, 1, 3, 7, 14, 21 and 28 after TBI. Significant injury and treatment effects were detected across all time points except day 0 (prior to injury). (Two-way repeated-measures ANOVA with Tukey’s multiple comparison test). Both vehicle and NALL-fed sham groups showed significant difference in motor function as compared to the TBI groups fed with NALL or vehicle in all time points except day 0 (^#^p < 0.0001). Significant improvement in motor function was detected in NALL-fed TBI group as compared to the vehicle-treated TBI mice on days 21 (**p < 0.01) and 28 (*p < 0.05). Data are presented as mean ± SEM, n = 10 Sham + vehicle, 8 Sham + NALL, 18 TBI + vehicle and 16 TBI + NALL. (**b**) Novel object recognition (NOR) test was performed on days 24 and 25 after TBI to assess non-spatial memory retention in vehicle or NALL-treated sham or TBI mice. Vehicle-fed TBI mice spent significantly less time with novel object as compared to NALL-treated TBI mice (*p < 0.05, Two-way ANOVA). Time spent with the novel object by the vehicle-treated TBI mice was also significantly less as compared to the vehicle (*p < 0.05) or NALL (**p < 0.01) -fed sham mice. No significant difference was observed between NALL-treated TBI and either of the sham groups. Data are presented as mean ± SEM, n = 10 Sham + vehicle, 9 Sham + NALL, 20 TBI + vehicle and 18 TBI + NALL. (**c**) Representative images of lesion volume at 28 days post injury in vehicle and NALL-treated mouse cortices. Images were acquired on Leica DM4000 B TL (BF) microscope and generated using Stereo Investigator software, 2020.2.3 version (MBF Biosciences) and representative black lesion volume outlines seen in the images were traced on Microsoft Powerpoint (Version 16.42). (**d**) Stereological (Cavalieri method) quantification of lesion volume in vehicle- or NALL-fed injured mouse cortices. Data are presented as mean ± SEM. n = 7/group, *p < 0.05 (Two-tailed Student’s t-test).

We assessed whether NALL treatment can attenuate impairments in motor and memory function in injured mice following TBI. We compared functional recovery between vehicle and NALL-treated TBI and sham mice using a battery of behavioral tests as depicted in Fig. 1a. We assessed motor co-ordination using beam walk test on days 1, 3, 7, 14, 21 and 28 post injury. We observed gradual improvement in motor function in NALL-treated TBI mice as compared to vehicle-fed TBI mice, starting from day 7 and reaching significance on days 21 (p = 0.0096) and 28 after injury (p = 0.012) (Fig. 6a). This demonstrates that NALL treatment can accelerate motor function recovery in TBI mice.

In order to assess effects of NALL on cognitive function after TBI, we used novel object recognition (NOR) tests. We performed novel object recognition test to assess non-spatial learning and memory. All TBI mice spent significantly less time with the novel object as compared to corresponding sham groups, confirming expected injury effect. However, NALL-fed TBI mice spent significantly (p = 0.0395; two-way ANOVA) longer time with the novel object as compared to the vehicle-fed TBI mice (Fig. 6b), suggesting improvement in memory retention following NALL treatment.

Finally, to determine whether NALL treatment could result in overall tissue repairing after TBI we used stereology to compare lesion volume between NALL and vehicle-treated TBI mice. Our data demonstrated significant decrease in lesion volume in NALL-fed TBI mice as compared to vehicle-fed TBI controls (Fig. 6c,d). Taken together, these results demonstrate attenuation of motor and cognitive deficits and decreased lesion volume in TBI mice fed with NALL as compared to the vehicle-treated TBI mice.

## Discussion

Repurposing of drugs that are already in clinical use for the treatment of certain medical conditions can be an effective and rapid way to develop useful therapeutic strategies for the treatment of highly devastating and intractable conditions like TBI. The racemic mixture of NAL has been used orally for more than 60 years in France to treat acute vertigo and dizziness^28^. It is also shown to be effective for the treatment of lysosomal storage disorders like NPC and GM2 Gangliosidosis (Tay-Sachs and Sandhoff disease) and the prophylactic treatment of migraine^16,18,19,29–31^. It is considered a safe drug as it does not show any major side effects at its therapeutic dose^19^. Recently, its l-enantiomer form (NALL) has been identified as the active form of NAL for disease-modification and neuroprotection^16^. In the current study using this active l-isoform we demonstrated neuroprotective function of NALL in the experimental TBI model of mice. This suggests therapeutic potential of NALL and provides the scientific basis for using NALL to treat TBI.

Early inhibition of cell death is very important in managing the devastating neurological outcome following TBI as markers of cell death peak at the acute time points following TBI, specifically in neurons^23^. We observed marked attenuation of cell death both in cortices and in hippocampi during the acute phase at day 1 after TBI in mice that were treated orally with NALL. Our data suggest that this might be mediated through the activation or restoration of autophagy flux by NALL. Autophagy has a neuroprotective function^32^ by maintaining cellular homeostasis and removing pathogenic protein aggregates and damaged organelles that have particularly deleterious effects in post-mitotic cells like neurons^25–27^. Impairment in autophagic function has been well documented in many neurodegenerative diseases including Alzheimer’s disease and Parkinson’s disease as well as lysosomal storage disorders like NPC and GM2^32–34^. We also previously demonstrated that autophagy is inhibited in the mouse cortex after TBI due to impaired lysosomal function leading to the accumulation of autophagosomes and toxic ubiquitinated proteins that contribute to neuronal death in the injured cortex^23,24^. Our and others’ data have also demonstrated that increasing autophagy flux after TBI has neuroprotective effects. Thus, early restoration of autophagy flux at day 1 after TBI by NALL treatment clearly demonstrates its beneficial effects in improving neuronal autophagy in the injured mouse cortices and thus suggests its therapeutic benefits in restricting progressive neuronal death after TBI. We expect similar mechanisms may contribute to disease-modifying benefits of NALL in other disorders where autophagy defects are observed.

NALL treatment also reduced neuroinflammation in the injured cortices of TBI mice. Acute and prolonged neuroinflammation contributes to the brain damage after TBI^9,10^. Pro-inflammatory cytokines secreted by activated microglia/macrophages following TBI promote neurotoxicity either by acting directly on neurons or by activating other glial cells including astrocytes that secret neurotoxic modulators^9,10^. Marked decline in proinflammatory cytokines like IL-1β in the injured cortices of TBI mice fed with NALL clearly suggested attenuation of pro-inflammatory activation by NALL treatment. Recently, detrimental effect of IFN-β mediated neuroinflammation has been demonstrated to cause chronic neurodegeneration in experimental TBI^35^. Thus, significant decrease in *Ifnb1* gene expression in the injured cortices of TBI mice following NALL treatment further demonstrates the beneficial effects of NALL treatment in TBI. Additionally, inflammatory response is also associated with the increase in oxidative stress. Previous studies have demonstrated that NOX2 activation can lead to increased ROS production^36–38^. Thus, the observed decrease in *Nox2* expression following NALL administration may be particularly significant as its genetic deletion or pharmacological inhibition is neuroprotective after TBI^37^. While NALL administration results in changes in anti-inflammatory genes *Arg1* and *Socs3* expression levels following brain injury, these levels gradually normalized to the levels observed in vehicle-treated TBI mice. Thus, NALL administration does not result in any long-term imbalance in anti-inflammatory responses which play important role in tissue repair after TBI. Together our data suggest that early attenuation of neuroinflammation is responsible for additional neuroprotective function of NALL after brain trauma.

Early attenuation of neurodegeneration and neuroinflammation in injured mice treated with NALL was associated with improved functional recovery and smaller lesion volume for up to 28 days after injury. The improvements in both motor and cognitive function clearly support a therapeutic potential of NALL in TBI. Improvements in motor function following NALL treatment in cerebral ataxia^11^ and in neurodegenerative lysosomal storage diseases like Niemann Pick^17,19^ and GM2 Gangliosidosis (Tay-Sachs and Sandhoff^30,31^) diseases have been reported previously. It has also been demonstrated that *N*-acetyl-leucine restores, prevents and delays disease progression in multiple neurological circuits of the brain, the clinical manifestation of which can be visualized and captured in terms of improvements or stabilization in very different processes such as ambulation, fine motor skills, speech, but also cognition^16,19,39^. The observed attenuation of memory deficit and improvement in cognition in TBI mice treated with NALL in this study supports the beneficial role of NALL treatment on cognitive function. Since TBI is a major risk factor for dementia, NALL-mediated improvement in memory function in injured mice indicates that it might be beneficial in attenuating injury caused dementia, although further long-term studies will be necessary. It will also be necessary to perform similar studies on female mice to determine if NALL treatment after TBI can yield similar results.

Our study clearly demonstrates therapeutic potential of NALL in attenuating neurological deficits, restricting neuronal loss and neuroinflammation after TBI and provides the scientific basis for the use of NALL as a TBI treatment.

## Methods

### Controlled cortical impact

All surgical procedures and animal experiments were performed as per the protocols approved by the Animal Care and Use Committee of the University of Maryland. All methods were performed in accordance with the guidelines and regulations of the Animal Care and Use Committee of the University of Maryland and as per the ARRIVE guidelines 2.0. Controlled cortical impact (CCI) induced TBI was performed in male *C57BL6/J* mice (20–25 g)^40^. We chose to use male mice only because in humans males have 1.5 time higher incidence of TBI than female^41^ and the neuropathological events after TBI are affected by female hormones^42^. Thus, in order to reduce variability of experimental outcomes and number of animals to reach statistical significance only male mice were chosen for this study. Briefly, after a 10-mm midline incision was made over the skull, the skin and fascia were retracted, and a 4-mm craniotomy was made on the central aspect of the left parietal bone of mice under surgical anesthesia (2–3% isoflurane evaporated in a gas mixture containing 70% N_2_O and 30% O_2_). Moderate injury was induced in the exposed brain by a custom microprocessor-controlled and compressed air driven pneumatic impactor of 3.5-mm diameter tip with the impact velocity of 6 m/s and a deformation depth of 2 mm. Following injury, mice were kept in a recovery chamber where temperature is maintained at 85° F and monitored continuously until they were able to maintain sternal recumbency then intermittently until they were ambulatory. Following that, animals were monitored regularly.

### NALL treatment

*N*-Acetyl-l-Leucine (441511, Sigma) was dissolved in ethanol to prepare 50 mg/ml solution which was then diluted in water to get 10 mg/ml. Since the food intake decreases in mice early after TBI^43^ and cell death and neuroinflammation peak at days 1 and 3 respectively following brain injury in mice^23^, NALL was given to the mice via oral gavage for 4 days after CCI-induced TBI so that the effective therapeutic concentration of NALL can be maintained in mice early after injury. Around 0.25 ml of NALL solution (10 mg/ml) was orally administered in mice at 100 mg/kg/day dose (2.5 mg NALL/25g mouse) via oral gavage, as described previously, starting at 1 h after CCI-induced TBI and continued once daily for 4 days. NAL has been shown to be safe even upon long-term administration (up to several months) and to improve outcomes in animal models in neurodegenerative diseases when administered IV at 10–15 mg/kg^15^. Because of the lower bioavailability of NALL when given orally (10–15%), 100 mg/kg of NALL was administered in mice per day orally to maintain its effective concentration. Oral administration was chosen because this route of administration is more clinically relevant and has been shown safe and effective in human patients and laboratory animals. Mice were also fed with NALL at 0.5 g/kg of chow for up to 28 days after injury (Fig. 1a). This was determined based on the average food intake of mice which is between 17 and 23% (≈ 20% average) of their own body weight per day^43,44^ that equivalents to 0.5 g NAL/kg chow powder.

### Western blot analysis

Around 5 mm tissue of ipsilateral cortex around the site of injury from TBI mice (1, 3 and 7 days post-TBI) or the corresponding tissue of same volume around the same cortical region from sham mice were dissected and processed as described^23^. Tissue lysates were resolved on 4–20% SDS-PAGE gels (Bio-Rad, 5671095) and transferred to PVDF membrane (Millipore, IPVH00010) using semi-dry transfer (Bio-Rad), blocked with 5% nonfat milk in tris buffered saline with 0.05% tween 20 (TBST), probed with primary antibodies in 1% BSA in TBST overnight at 4 °C and incubated with HRP-conjugated secondary antibodies (KPL, 474-1506, 474–1806, 14-16-06 and 14-13-06) in blocking solution at room temperature for 1 h. Protein bands were detected using chemiluminiscence kit (Pierce, 34076) and visualized using Chemi-doc system (Bio-Rad). Band intensity was analyzed using Image Lab software (Bio-Rad) and normalized to loading control (β-Actin). For western blot analysis n = 5 mice/group, and 3 samples shown in representative Figs. 2a and 3a.

Primary antibodies: LC3 (1:1000; Novus, NB100-2220), p62/SQSTM1 (1:1000; BD Bioscience, 610832), β-actin/ACTB (1:10,000; A1978) and fodrin/spectrin (1:5000; Enzo Life Science International, BML-FG6090). Uncropped images of western blots were provided in the Supplementary Figs. S1 and S2.

### TUNEL assay

20 μm frozen sections were obtained from vehicle or NALL-fed sham and TBI mouse brains (n = 6/group and 4 sections/mouse) at day 1 post-TBI, following fixation with 4% paraformaldehyde (PFA, pH 7.4) and protection in 30% sucrose, as previously described (27). TUNEL -positive cells were detected in NALL-fed sham and TBI at 1 day post-TBI, using ApopTag In Situ Apoptosis Detection Kit (Millipore, S7165) as per the manufacturer's protocol. Images of TUNEL positive cells in the cortical region proximal to the injury site were acquired using a fluorescent Nikon Ti-E inverted microscope, at 20X (CFI Plan APO VC 20X NA 0.75 WD 1 mm) and quantified using Nikon Elements software (V4.12.01, Nikon) as described previously^23,24^.

We quantified the number of TUNEL + cells relative to the total number of cells counted. The average number of total cells counted/mouse was approximately 4000 cells/mouse. After the number of TUNEL + cells were normalized to the total number of counted cells, they were then expressed as TUNEL + cells/100 cells.

### Immunohistochemistry

20 μm frozen sections were obtained from vehicle or NALL-fed sham and TBI mouse brains at 1 day post-TBI (n = 5/group) following fixation with 4% paraformaldehyde (PFA, pH 7.4) and protection in 30% sucrose, as previously described (27). Sections were blocked with 5% goat serum (Millipore, S30-100) in 1(×) phosphate-buffered saline (PBS; Quality Biological, INC., 119-069-101) containing 0.025% Triton X-100 (Sigma, T8787), incubated. Sections were incubated overnight with primary antibodies at 4 °C and then with secondary antibodies in the blocking solution for 2 h at room temperature. Nuclei were stained with DAPI. 20 × images of the immune-stained sections were acquired using Nikon Eclipse Ti-E/Ni-E microscope and analyzed and quantified by Elements software (V4.12.01, Nikon).

Primary antibodies used include: SQSTM1 (1:200; Progen, GP62-C), LC3 (1:250, Novus Biologics), NeuN (1:500; Millipore, MAB377), cleaved caspase-3 (1: 200; Cell Signaling Technology, 9661). Secondary antibodies: Alexa Fluor 488 goat anti-rabbit (A11034), Alexa Fluor 633 goat anti-guinea pig (A11075), Alexa Fluor 633 goat anti-mouse (A21052).

We quantified the number of p62+ cells relative to the number of total cells counted. The average number of total cells counted/mouse was approximately 4000 cells/mouse. Once the number of p62+ cells were normalized to the total number of counted cells, they were then expressed as p62+/100 cells. For cleaved caspase-3 and NeuN double staining, the quantification was expressed as number of cleaved caspase-3 + neurons/100 neurons.

### Real time PCR

Around 5 mm tissue of ipsilateral cortex around the site of injury from TBI mice (1 day, 3 day and 7 day post-TBI) or the corresponding tissue of same volume around the same cortical region from sham mice were dissected and processed as described^23^. Total RNA isolated using miRNeasy Mini Kit (Qiagen, Cat No. 217004) was converted into cDNA using High Capacity RNA to cDNA kit (Applied Biosystem, Cat. No. 4387406) as per manufacturer's instruction. cDNA TaqMan Universal Master Mix II (Applied Biosystems, Cat. No. 4440040) was used to perform quantitative real-time PCR amplification as described previously^23^ using 20 × TaqMan^®^ Gene Expression Assay (Applied Biosystems) for the following mouse genes: *Gapdh* (Mm99999915_g1), *Nlrp3* (Mm00840904_m1), *Cybb* (Mm01287743_m1), *Nos2* (Mm00440502_m1), *Tnf* (Mm00443258_m1), *Ifnb1* (Mm00439552_s1), *Il1b* (Mm00434228_m1), *Arg1* (Mm00475988_m1), *Socs3* (Mm00545913_s1), *Chil3* (Mm00657889_mH) and *Il4r* (Mm01275139_m1) (Applied Biosystems). Reactions were amplified and quantified by using a 7900HT Fast Real-Time PCR System and corresponding software (Applied Biosystems). Relative gene expression normalized to *Gapdh* was calculated based on the comparative Ct method^45^. For this study n = 5/group for all time points.

### Behavioral methods

A battery behavioral tests were performed at time points depicted in Fig. 1a. All functional assessment and behavioral tests were performed and scored blinded.

#### Beam walk test

Motor co-ordination was assessed in sham and vehicle or NALL-fed TBI mice using beam walk test performed on days 0 (before injury), 1, 3, 7, 14, 21 and 28 after TBI as described previously^24,40^.

#### Novel object recognition (NOR) test

Hippocampal spatial memory was measured on days 24 and 25 after TBI using NOR test as per the method described previously^24,40^. Relative time spent with the novel object over total time spent with novel and familiar object is expressed as discrimination index.

### Lesion volume

Cortical lesion volume was assessed in vehicle and NALL-treated injured mouse brains at day 28 after TBI. 60 μm sections located approximately 240 μm apart across the entire lesion volume were stained with cresyl violet (FD Neurotechnologies Inc) and images were acquired on Leica DM4000 B TL (BF) microscope. Quantification was based on the Cavalieri method using Stereo-investigator software**, **version 2020.2.3 (MBF Biosciences) and the lesion volume was quantified by outlining the missing tissue on the injured hemisphere using the Cavalieri estimator with a grid spacing of 0.1 mm.

### Statistics

All data are presented as mean ± standard error of the mean (SEM). One-way ANOVA or two-way ANOVA was performed followed by appropriate post-hoc test as specified in the figure legends. For data with only two groups 2-tailed student t-test with equal variance was used. Statistical analyses were performed using GraphPad Prism program, version 3.02 for Windows (GraphPad Software, San Diego, CA, USA). A p value ≤ 0.05 was considered statistically significant. Two-way ANOVA with repeated measure was performed for beam walk analysis. Behavioral and stereological analyses were performed by an individual who was blinded to injury or treatment groups. Number of animals used in this study was determined by Power analysis (power of 0.8; alpha value 0.05). Mice were distributed randomly into different groups and time points.

## Supplementary Information

Supplementary Information.
