# Supplementary figures and images for: N-Acetyl-l-leucine improves functional recovery and attenuates cortical cell death and neuroinflammation after traumatic brain injury in mice
Source: Sci Rep. 2021 Apr 29;11:9249. doi: 10.1038/s41598-021-88693-8 (PMC8084982; doi:10.1038/s41598-021-88693-8)

Figure S1

**a**

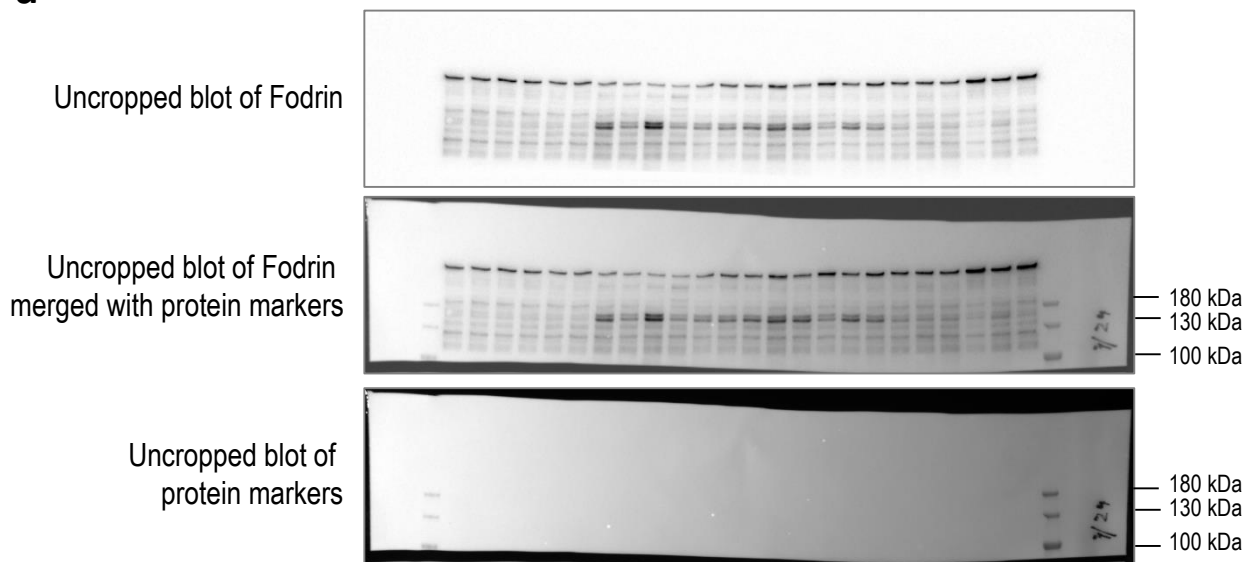

**b**

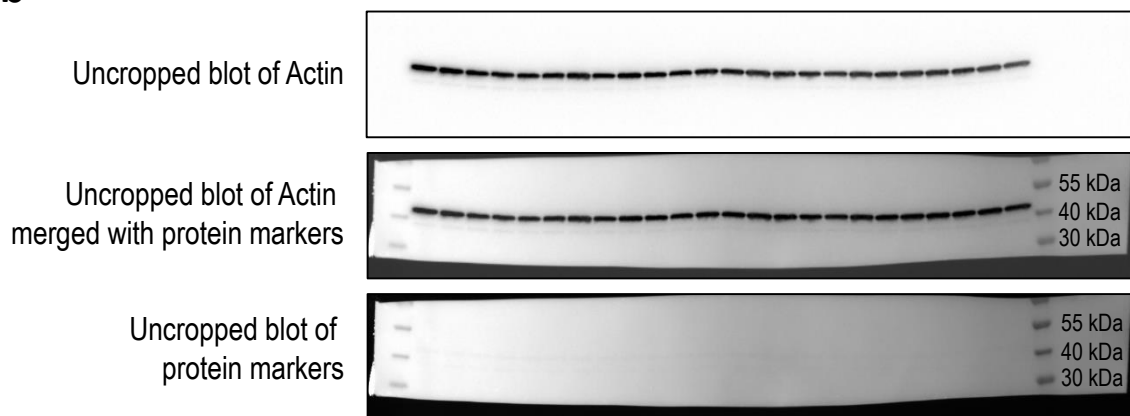

Figure S2

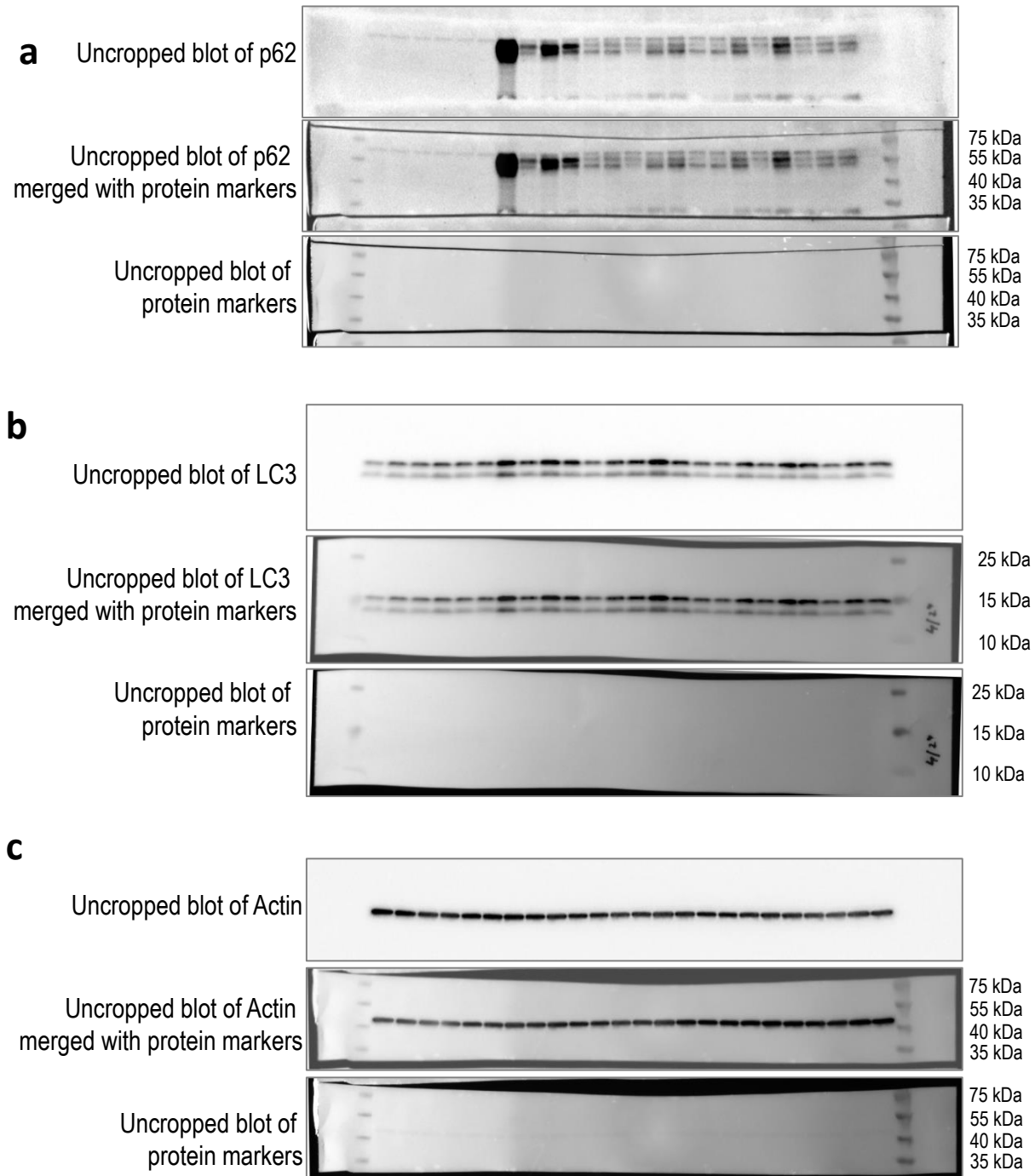

Supplement: Supplementary file 1 — Supplementary Information. [file 41598_2021_88693_MOESM1_ESM.pdf]
